# Deterministic 3D Ray-Launching Millimeter Wave Channel Characterization for Vehicular Communications in Urban Environments †

**DOI:** 10.3390/s20185284

**Published:** 2020-09-16

**Authors:** Fidel Alejandro Rodríguez-Corbo, Leyre Azpilicueta, Mikel Celaya-Echarri, Peio Lopez-Iturri, Imanol Picallo, Francisco Falcone, Ana Vazquez Alejos

**Affiliations:** 1 School of Engineering and Sciences, Tecnologico de Monterrey, Monterrey 64849, Mexico; alek.bsb@gmail.com (F.A.R.-C.); mikelcelaya@gmail.com (M.C.-E.); 2 Electrical, Electronic and Communication Engineering Department, Public University of Navarre, 31006 Pamplona, Spain; peio.lopez@unavarra.es (P.L.-I.); imanol.picallo@unavarra.es (I.P.); francisco.falcone@unavarra.es (F.F.); 3 Institute of Smart Cities, Public University of Navarre, 31006 Pamplona, Spain; 4 Signal and Communications Theory Department, University of Vigo, 36310 Vigo, Spain; analejos@uvigo.es

**Keywords:** millimeter wave, ray tracing, vehicular communications, channel characterization

## Abstract

The increasing demand for more sensors inside vehicles pursues the intention of making vehicles more “intelligent”. In this context, the vision of fully connected and autonomous cars is becoming more tangible and will turn into a reality in the coming years. The use of these intelligent transport systems will allow the integration of efficient performance in terms of route control, fuel consumption, and traffic administration, among others. Future vehicle-to-everything (V2X) communication will require a wider bandwidth as well as lower latencies than current technologies can offer, to support high-constraint safety applications and data exhaustive information exchanges. To this end, recent investigations have proposed the adoption of the millimeter wave (mmWave) bands to achieve high throughput and low latencies. However, mmWave communications come with high constraints for implementation due to higher free-space losses, poor diffraction, poor signal penetration, among other channel impairments for these high-frequency bands. In this work, a V2X communication channel in the mmWave (28 GHz) band is analyzed by a combination of an empirical study and a deterministic simulation with an in-house 3D ray-launching algorithm. Multiple mmWave V2X links has been modeled for a complex heterogeneous urban scenario in order to capture and analyze different propagation phenomena, providing full volumetric estimation of frequency/power as well as time domain parameters. Large- and small-scale propagation parameters are obtained for a combination of different situations, taking into account the obstruction between the transceivers of vehicles of distinct sizes. These results can aid in the development of modeling techniques for the implementation of mmWave frequency bands in the vehicular context, with the capability of adapting to different scenario requirements in terms of network topology, user density, or transceiver location. The proposed methodology provides accurate wireless channel estimation within the complete volume of the scenario under analysis, considering detailed topological characteristics.

## 1. Introduction

The concept of Smart City is so broad and relatively immature that its scope is not yet fully defined. The main motivation behind this concept comes from the efficient use of the available resources [1]. The implementation of the triad computing, communication and sensing can increase the efficiency levels significantly in several areas such as hydraulic resources, waste management, crime control, traffic management and transportation, among others. Within the different notions of what a Smart City should provide, Intelligent Transportation Systems (ITS) emerge as a promising idea to increase efficiency in fuel use, gas emission, routes optimization and drivers’ safety [2].

In a world society where approximately 1.35 million people die annually in a traffic-related accident, which is the eighth leading cause of death for people of all ages and the first for people between 5 to 29 years old [3]; the use of ITSs could play an important role towards the solution of this critical problem. Its approach to solving this scourge is to make vehicles go beyond human deficiencies, which means vehicles with the ability to sense their surroundings and act accordingly.

In addition, the automotive companies are continuously increasing the number of sensors inside their cars to improve the user experience and increase the automation of the overall system. The rising need for electric vehicles for pollution control and the increasing trend towards the expectations of autonomous vehicles are the main demands that have ensured high growth in the development and the introduction of more sensors inside the vehicles. In fact, the automotive sensor market was valued at $13,293.2 million in 2017 and is projected to reach $30,858.5 million by 2025, growing at a compound annual growth rate (CAGR) of 11.1% from 2017 to 2025 [4].

The integration of these sensors will support advanced driver assistance systems (ADAS). The next generation of autonomous vehicles that will assist or completely replace the driving system in vehicles will be implemented on top of this technology. As a result, there has been extensive research on ADAS systems in recent years, the variety of on-board sensors (e.g., radars, cameras, and LiDARs) are used to reduce the number of traffic accidents and deaths [5]. Although ADAS applications are still in the early stages, they will transform the driving experience and simultaneously provide road safety. ADAS will soon replace the humans behind the wheel of the car, reducing the number of accidents and allowing cars to be driven efficiently. These assisted systems will require the use of robust high-precision and redundant sensors for their full operation. In this sense, in the automobile industry, semiconductor companies will play a key role in the development of these technologies. New trends for automated vehicle systems promise vehicular linking with cloud-based applications and wireless connections with their surroundings. The fusion of these sensors as a robust communication system will allow the implementation of systems such as adaptive cruise control, extension of operational area, collision warning or mitigation on moving and still objects, top view, pedestrian protection/recognition, traffic jam assist, intersection/turning assist, overtaking assistance and construction area assistance, which are key enablers in driverless cars [6].

It is in this context that the term vehicle-to-everything (V2X) communication is defined. This concept performs a key role in the ITS environment, being the way of sharing and collecting knowledge between intelligent elements (e.g., another vehicle, building, traffic light). V2X communication will pave the way to exchange information between the different applications of ITSs related to traffic safety and efficiency, automation driving, information, and entertainment (infotainment). The term includes but is not limited to, Vehicle-to-Vehicle (V2V), Vehicle-to-Network (V2N), Vehicle-to-Pedestrian (V2P), and Vehicle-to-Road Infrastructure (V2I) communication [7]. Other concepts used are Vehicle-to-Device (V2D), Vehicle-to-Grid (V2G), and vehicle to roadside units (RSU). RSUs are a particular type of V2I located on both sides of the avenue providing information and connectivity to nearby vehicles. Its applications include data disseminators, traffic records, security handlers, and service proxies.

Although the inclusion of large amounts of sensors involves the creation of smarter vehicles, the fields of action of these sensors have their limitations. The adoption of cooperative systems between vehicles on the route would allow the sharing of unprocessed or semi-processed sensor data between vehicles, allowing the system to obtain more accurate information and make smarter decisions [8]. This real-time exchange of sensor data requires high communication performance, and with the increase in vehicle density on the road many simultaneous connections will also be required [9]. Future autonomous vehicles will also generate large amounts of data in areas such as high-speed internet, video streaming, online video games, and real-time map information. The massive traffic of data produced by these sensors will require transfer rates in magnitudes of gigabits per second to communicate with other vehicles or road infrastructure. This capacity will allow bringing cloud computing to the era of autonomous driving [10]. Consequently, the demand for wireless techniques that support this high bandwidth has increased considerably in recent years. Wireless connectivity with high transfer rates is predicted to be the next frontier in the vehicular revolution.

Different technologies have been considered for the use of vehicular communication, some of these include Bluetooth, satellite radio, and communication through visible light. Despite these different technologies, the strongest candidates for the future of vehicular communication are the Dedicate Short Range Communication (DSRC) supported by the IEEE 802.11p standard and the use of the cellular mobile network (C-V2X) [11]. Although DSRC can ensure low-latency communication, its maximum transfer speed hardly exceeds 6 Mb/s in real applications. For C-V2X, higher transfer levels can be achieved, in practice usually values of 100 Mb/s, but communication latencies cannot get below 100 ms [12].

In recent years, significant academic activity, motivated by these requirements, has proposed the adoption of mmWave frequencies (i.e., 10–300 GHz) to support high transfer rates and low latencies [13]. Although the use of mmWave bands promises a significant increase in data rates with less latency, its use comes with high constraints for implementation. Free-space losses are very high at these frequencies, as a result, communication ranges will decrease considerably. The poor diffraction associated with these frequency ranges produces a more aggravated shadowing effect, this associated with the poor signal penetration, leads to deep losses due to objects in the communication path [14]. Wireless links at these high frequencies suffer from many deteriorating factors which pose challenges to the V2X communication system. In addition to these limitations, the high medium mobility and the relatively low height of the transceivers also increases Doppler effect phenomenon and occurrences of non-line-of-sight (NLOS) links. Due to its direct relationship with the relative speed of the transceivers, the Doppler effect is one of the most critical concerns in the use of mmWave in vehicular communications.

The V2X propagation channel exhibits rapid temporal variability, and due to the dynamics of its physical environment, the statistics of the channel exhibit a non-stationary behavior, which makes the design of V2X systems in the mmWave frequency bands a challenging task. Further studies in these frequency bands are needed to understand the unique characteristics of vehicle communications, including gas attenuation, rain absorption, increased scatterers and reflections due to poor diffraction, and high penetration losses related to these frequency bands [9]. The International Telecommunication Union (ITU) and the World Radiocommunication Conference (WRC) have identified different mmWave frequency bands for terrestrial systems delivering high-throughput, low-latency 5G services [15]. These bands are: 24.25–27.5 GHz (24 GHz), 37–43.5 GHz, 45.5–47 GHz, 47.2–48.2 GHz, and 66–71 GHz. The V-band of 60 GHz is not in the above list given that it is reserved for the WiGig standards WiFi 802.11ad and WiFi 802.11ay [16,17,18,19]. The 5G Phase 2 or Beyond 5G also contemplates V2X communications as use cases in mmWave frequency bands. This is due to the fact that cellular and WiGig current standards are not enough for the requirements of the specific types of V2X applications which demand extreme connectivity performance: ultra-low latency (less than 1 ms delay), high security, ultra-reliable connectivity for safety applications, and enhanced mobile broadband in order to provide augmented reality (data rate over 1 Gb/s) [20,21]. Only mmWave bands can support ultra-high-speed mobile broadband demanded for 5G automotive use case [22].

The use of mmWave bands for vehicular communication is particularly attractive for future applications of ITS. The use of directional antennas and multiple-input multiple-output (MIMO) systems will require beam alignment, in a vehicular environment with high mobility, the appropriate design for beam tracking and prolonged link establishment is a highly complex task. The reduction in the communication radius will be subject to regular handovers, and due to the constant change of the communications channel as well as the sudden appearance and disappearance of scatterers, there will also be high restrictions in time to perform such handovers. Identity authentication and establishing a trusted connection with the cloud will be a difficult task in a V2X network [23]. The abstraction of the characteristic parameters in the electromagnetic transmission channel towards propagation models can shed light on the complex propagation mechanisms in mmWave bands and allow the evaluation of the designed communication systems [24]. Nevertheless, due to the propagation differences between micrometer waves and mmWave, the propagation models used in vehicular environments for under 6 GHz systems cannot be used in scenarios for mmWave frequency bands. Thus, achieving effective communication in mmWave links for vehicular environments requires a deep understanding of the vehicle communication channel. This calls for new models that account for the specific characteristics of communications in the millimeter frequency bands.

To this end many propagation models has been developed in the literature, taking into account the availability or not of geometric information of the environment. In this sense, the main approaches can be classified as deterministic or stochastic geometric models and stochastic or empirical non-geometric models. Empirical models are based on approximations resulting from the collection of experimental measurements, they are simple, relatively effective, and accurate, for environments that resemble some of the characteristics in which the measurements were made. The input parameters for the empirical models are usually very general (e.g., urban area, rural area). One of the main disadvantages of these models is that they must be employed in environments similar to those used in their estimation, requiring strong modifications for their use in other environments, and in many cases, they are simply ineffective outside of these scenarios [25]. Some empirical studies regarding sub 6 GHz vehicular propagation channels can be found in the literature like [5,26,27]. Non-geometric stochastic models (NGSCM) are used to predict the values regarding the modeled system, taking into account the occurrence of random elements within the system. They are designed to simulate uncertainty in different scenarios. These models can be relatively versatile to describe propagation channels in different environments. Some V2X non-geometric stochastic models can be found in [28,29] for the 5–6 GHz band.

If the spatial distribution of the environment is available, geometric stochastic models (GSCM) can be implemented using a simplified distribution of scatterers around the transceivers to identify and emulate the statistics of the physical environment. Some recent propagation models under this category can be found in [30] for mmWave bands and for sub 6 GHz in [31,32]. Another geometric approach is the use of deterministic propagation models, this kind of modeling technique characterizes the communication channel using detailed scenario information. They are site-specific methods and are generally very exhaustive at a computational level. Ray Tracing (RT)/Launching (RL) is a deterministic approach that, at the expense of its high level of computational load, achieves a good tradeoff between accuracy and computational cost.

In this work, we present the analysis of an urban V2X mmWave link scenario using a deterministic in-house Matlab-based 3D ray-launching (3D-RL) algorithm and empirical data obtained throughout a measurements campaign [33]. The proposed methodology enables the obtaining, for the complete volume of the scenario under analysis, of estimation of frequency/power as well as time domain parameters. The main contributions of this work are the following:

- Full volumetric deterministic scenario simulation capabilities, which enable the analysis and evaluation of wireless system radio planning tasks, considering multi-link, multi-node configurations of any type.
- Estimation of frequency/power as well as time domain statistics for any given distribution of scatterers, considering frequency dispersive properties of all the elements within the scenario. This enables the analysis of multiple vehicular communication link types as a function of scenario as well as traffic conditions.
- Optimized simulation technique based on the use of hybrid simulation approach, based on the combination of 3D-RL with multiple acceleration techniques, in order to effectively consider large scenarios with topological detail levels.

**Figure 1:**
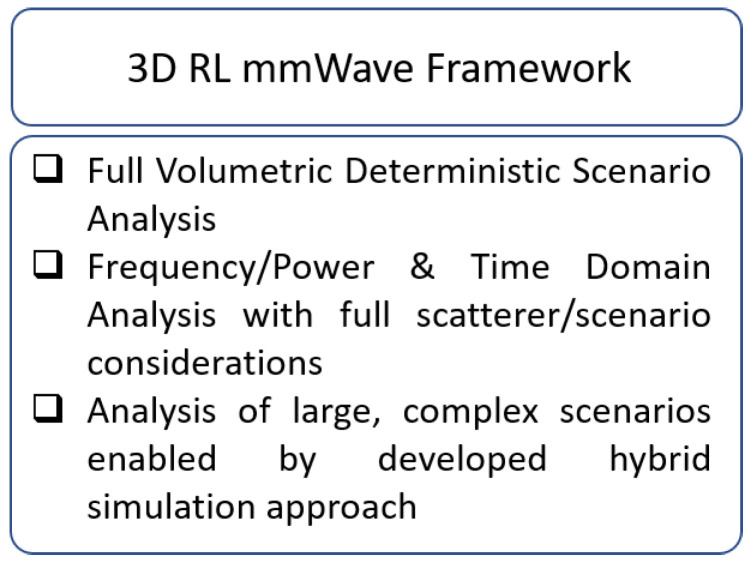
Schematic overview of the main contributions of the 3D-RL mmWave framework.

A schematic overview of the main contributions of this work is given in Figure 1. The 3D-RL analysis can be extended to any requirement in terms of network/node configuration, node density or user location. The manuscript is organized as follows: an overview of some relevant related investigations is presented in Section 2. Then, the proposed simulation technique and the scenario description is presented in Section 3, as well as the simulation parameters. Section 4 shows the outcomes of the simulation in terms of the received power, Power Delay Profile (PDP) and time dispersion parameters like the Root-mean-squared Delay Spread (RMS DS). Section 5 presents the measurements results, where all data collected in the real scenario is analyzed, and comparisons are drawn against the results obtained in the simulation stages. Finally, conclusions and the main takeaways are highlighted and summarized in Section 6.

## 2. Related Work

Wireless communications in the mmWave band promise an increase in transfer rates while decreasing communications latency. However, high transmission losses, and poor diffraction and signal penetration pose obstacles to the complete arrangement of mobile communication systems in these frequency bands. The complete understanding of all the characteristics behind the transmission in the mmWave frequency bands is fundamental for the application of these high-throughput wireless communication systems. This multi-gigabit communication will allow sharing raw sensor data between ITS elements. Despite this, its application in transport systems is currently limited to radar arrangements [13]. Although the use of mmWave as a communication system in the vehicular environment is a concept already established almost two decades ago [34], it is only recently that its practical application for these purposes seems more real. However, the empirical-based propagation models are not able to provide accurate space-time or angle-delay results, which are key characteristics for simulation of these systems. Using geometric methods allows the analysis of these systems natively, in which techniques such as RT/RL will be useful to address complicated propagation conditions in high-frequency regimes [35]. The use of these techniques for the analysis of wireless links at these frequency bands can be verified in works such as those shown in Table 1, where references like [36,37,38,39] make use of these approaches in a wide range of scenarios spanning indoor and outdoor environments.

Specifically, in vehicular environments, RT/RL is a valuable tool to analyze the wireless communication channel. In mmWave frequencies, works such as [40] use the RT technique to analyze aspects of vehicular communication under NLOS conditions. In such work, a professional RT tool called Wireless Insite is selected and the results gathered in simulations are compared with statistics obtained during measurement campaigns. The environment is staged under V2I communication conditions, and parameters such as Path Loss exponent, shadowing factor, and RMS DS are some of those obtained in the work. Another communication environment for transport systems is the high-speed trains (HST) scenarios that are rarely explored. The work presented by [41], uses a calibrated and validated 3D-RT algorithm to explore the characteristics of the 25.25 GHz wireless channel in HST scenarios. Multiple layouts are simulated, and parameters such as Path Loss, PDP, RMS DS, and Doppler effects are some of those obtained during RT simulations. In addition, another RT algorithm is used to characterize the influence of typical objects in the context of trains to infrastructure communication in the work conducted by [42]. The research carried out at 28 GHz, uses a calibrated algorithm and experimental measurement campaigns. Both simulation and the measurement campaign uses an omnidirectional antenna and a Close-in (CI) free-space reference distance model to fit the data. Path Loss parameters and RMS DS are obtained in different scenarios, which are expanded with the assistance of RT simulations. In [43], the wireless communication channel is characterized in a V2I environment in the mmWave frequency bands. The work is also assisted by a RT algorithm, validated by experimental measurement campaigns at 60 GHz, and then used in 22.6 GHz simulations. Through the comparison of parameters such as received power and RMS DS, the algorithm precision is validated. As part of the conclusions drawn in such research, a relationship is found between the RMS DS and traffic flow density, highlighting that this factor increases when the traffic density rise, related to the addition in the number of multipath generated by the vehicles. Another work presented in [44], based on the characterization of the wireless channel in a V2I scenario, also employs the assistance of a deterministic technique. The study is performed at a frequency of 28 GHz under an omnidirectional pattern and implements simulations to obtain the Path Loss parameters. The infrastructure is identified as an RSU located at 5 m height. The data is fitted to a CI free-space model, also used in research previously reported as [40,42]. Further studies have been performed in order to analyze and enhance wireless channel operation, by analyzing interference impact, the use of deep learning techniques to optimize beam forming parameters or the proposal of new effective receiver volume configurations, among others [45,46,47,48,49].

In this work, compared to the previously presented works, the wireless communication channel in a vehicular environment is analyzed in the mmWave frequency bands (28 GHz), with the aid of an in-house developed 3D-RL algorithm, a powerful tool to expand the scarcity of empirical data in these frequency bands. In comparison with [42], together with [41], they apply a RT technique in HST communication scenarios, a completely different environment from the urban vehicle setup presented in this work. In the case of [40,43,44], these studies are dedicated to V2I communication in different conditions. In the work carried out in [50], the communication channel is modeled for a V2V environment with the inclusion of obstructions, but the research is carried out in the 5 GHz band. Comprehensive surveys in relation to vehicular communications can also be found in [51,52]. However, the presented work aims to increase the knowledge about the wireless communication channel at mmWave bands, with special focus on the impact of different obstructions in the V2V communication links, and its effects on large and small-scale fading parameters. The proposed methodology can be further extended with no loss of generality to multiple studies, such as interference analysis or coverage/capacity estimations, for different scenarios and applications.

## 3. Materials and Methods

### 3.1. Ray-Launching Simulation Technique

With the aim of analyzing the impact of the different channel impairments present in an urban V2X communication link at 28 GHz frequency band, an in-house developed 3D-RL has been used. The algorithm has been widely used and validated for urban complex scenarios and V2X links for frequency bands below 6 GHz. For instance, in [53], an urban environment is simulated by means of the in-house 3D-RL algorithm, coupled with a microscopic vehicular movement simulator, showing good agreement with experimental measurements for the frequency band of 5.9 GHz. On the other hand, in [54], with the aid of the 3D-RL tool, the radio wave characterization for ISM 2.4 GHz and 5 GHz Wireless Sensor Networks (WSNs) deployed within a smart city scenario has been assessed and validated. It has also been validated for wireless propagation in closed environments [55], interference analysis [56] or electromagnetic dosimetry evaluation in wireless systems [57]. A detailed description of the algorithm and its convergence analysis can be found in [58]. In this work, a further step is proposed, to analyze the impact in the physical layer of the frequency band of 28 GHz, which arises as a potential band for the next generation of vehicular communications.

The in-house developed 3D-RL is based on Geometrical Optics (GO) and the Uniform Theory of Diffraction (UTD). The 3D-RL approach basis consist to launch a significant set of rays that travel from the transmitter to the receiver position considering the direct ray (LOS), reflected and diffracted rays according to the geometry and morphology of the considered scenario. The whole channel information is achieved by means of the resulting complex impulse response. However, to obtain accurate results, a thorough description of the site-specific propagation scenario is required. The application of GO can cause some channel prediction impairments in edges and discontinuities areas, which are resolved in the algorithm with the consideration of the diffraction phenomenon based on UTD. Thus, the diffraction coefficients on the obstacles edges or diffractive elements have been considered to predict the shadowing areas’ field. The electric field E created by GO and the diffracted electric field created by UTD are calculated by [59].

(1) $$E_{GO}^{⊥\|}=\sqrt{\frac{P_{rad}D_{t}\left(\theta_{t},\phi_{t}\right)\eta_{0}}{2\pi }}\frac{e^{-j\beta_{0}r}}{r}X^{⊥\|}L^{⊥\|}$$

(2) $$E_{UTD}^{⊥\|}=e_{0}\frac{e^{-jks_{1}}}{s_{1}}D^{⊥\|}\sqrt{\frac{s_{1}}{s_{2}\left(s_{1}+s_{2}\right)}}e^{-jks_{2}}$$

where $\beta_{0}=2\pi f_{c}\sqrt{\epsilon_{0}\mu_{0}}$, $\epsilon_{0}=8.854\times 10^{-12}$ F/m, $\mu_{0}=4\pi 4\times 10^{-7}$ H/m and $\eta_{0}=120\pi$ ohms. The radiated power of the transmitter antenna is denoted as $P_{rad}$. $D_{t}\left(\theta_{t},\phi_{t}\right)$ is the directivity where rays are launched as defined in the spherical coordinate system at an elevation angle $\theta_{t}$ and an azimuth angle $\phi_{t}$. $X^{⊥\|}$ and $L^{⊥\|}$ are the polarization ratio and path loss coefficients for each polarization, *r* the distance in free space and fc the transmission frequency. In Equation (2), $D^{⊥\|}$ are the diffraction coefficients for each polarization, $e_{0}$ is the free-space field strength, *k* is the propagation constant and $s_{1}$, $s_{2}$ are the distances from the source to the edge and from the edge to the receiver point.

**Figure 2:**
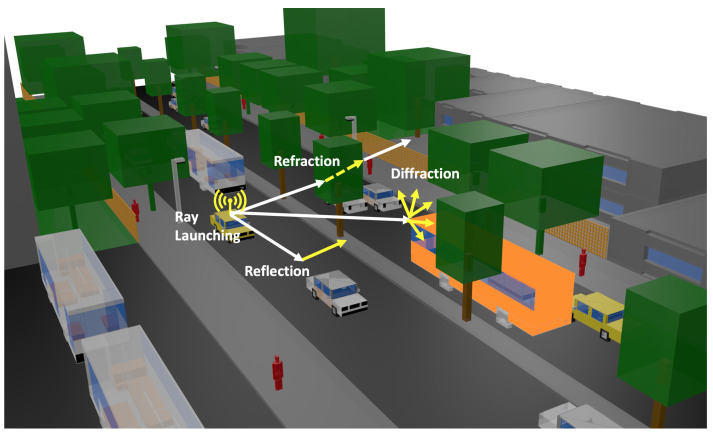
Schematic overview of the operation principle of the 3D-RL software.

The launching rays are predefined in a solid angle that considers the radiation diagram of the transceivers. Parameters such as frequency of operation, radiation patterns of the antennas, number of multipath reflections, separation angle between rays, and cuboid dimension can be considered to be inputs in the 3D-RL software. Moreover, all the material properties for all the elements within the scenario can also be considered, given the conductivity and relative permittivity of all the obstacles at the frequency range of the system operation under analysis. Figure 2 presents a schematic overview of the 3D-RL principle in the considered scenario, where reflection, refraction and diffraction are depicted in the vehicular scenario. The detail of the different sizes of vehicles can also be observed.

**Figure 3:**
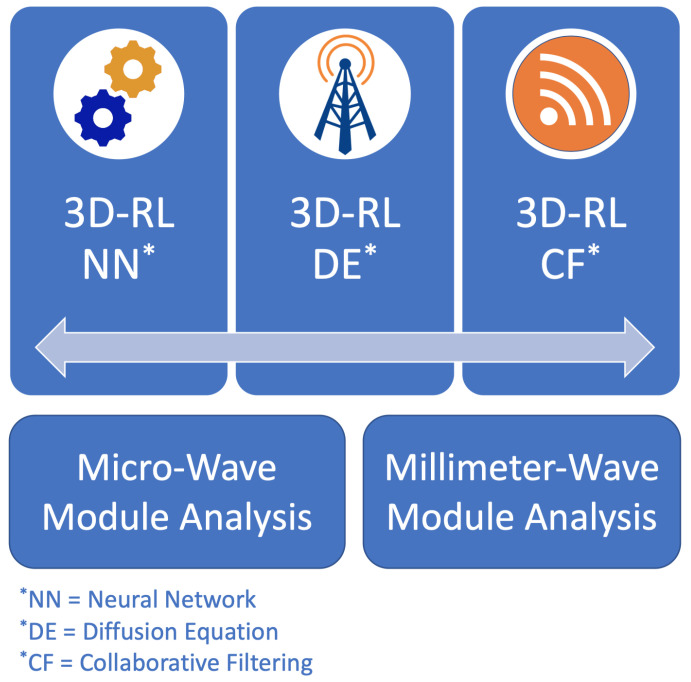
Schematic view of the 3D-RL implemented modules with the combination of hybrid techniques to reduce the computational load. Different material properties and propagation phenomena are considered in the different modules based on the frequency under analysis.

As presented in the literature, the principle of GO/UTD foresees precisely wireless communication propagation when a complex 3D scenario is considered [60], with the principal disadvantage of a high computational cost. In this sense, in order to overcome this drawback, different hybrid techniques have been proposed combining the 3D-RL algorithm with different approaches, such as Neural Networks (NN) [61], Diffusion Equation (DE) [62] or Collaborative Filtering (CF) [63]. These hybrid techniques achieve accurate results while reducing the computational cost for complex scenarios. The 3D-RL tool is based on a modular structure, where different libraries can be integrated. Thus, to consider the frequency band of 28 GHz, the 3D-RL tool has been updated with the mmWave module. Figure 3 presents the schematic view of the in-house developed simulation algorithm for propagation prediction at micro-wave and mmWave frequency bands, integrated with the different hybrid techniques to reduce the computational load for complex scenarios.

In the implementation of the mmWave module, the different electric conductivity and relative permittivity models for the different materials within the scenario for the 1–100 GHz frequency range, obtained by the Recommendation ITU-R P2040-1 [64], have been added. In addition, the capability of using beamforming for the transceivers has been included, to emulate real V2X scenarios for these frequency bands. The analysis in different beam directions can be generalizable for all the possible vehicular situations in the in-house 3D-RL proposed simulation tool, as well as different angular characteristics, as presented in [65]. Moreover, the proposed methodology enables the characterization of different conditions and new case studies, such as indoor/outdoor transitions or variable height link usage, which can be given for vehicle considerations or the use of diverse urban infrastructure to allocate transceivers. Finally, the atmosphere absorption phenomena have also been considered, which is more relevant at higher frequencies [66].

### 3.2. Scenario Description

**Figure 4:**
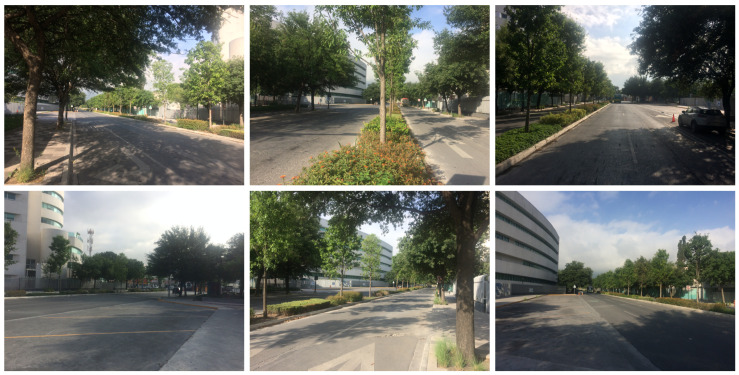
Real scenario collage of photos with considered scatterers as buildings, lamp posts, fences, and vegetation, among others.

In this section, the real scenario used to model vehicular communication links through the 3D-RL algorithm is described as well as its rendered schematic view used for simulation. Figure 4 presents a collage of photos of the real scenario, located in a side street of Tecnológico de Monterrey, Campus Monterrey, Mexico. As depicted, the modeled fragment corresponds to an area of approximately 150 × 68 m with several types of vegetation, such as different trees and shrubbery. The portion corresponding to the avenue occupied by the vehicles has a length of 150 m and a width of 16.5 m, where a median strip with inhomogeneous vegetation and two lanes per driving direction are included, representing a classical urban complex heterogeneous vehicular scenario. Moreover, abundant vegetation, private fences and buildings with one to eight floors are surrounding the delimited segment area.

**Figure 5:**
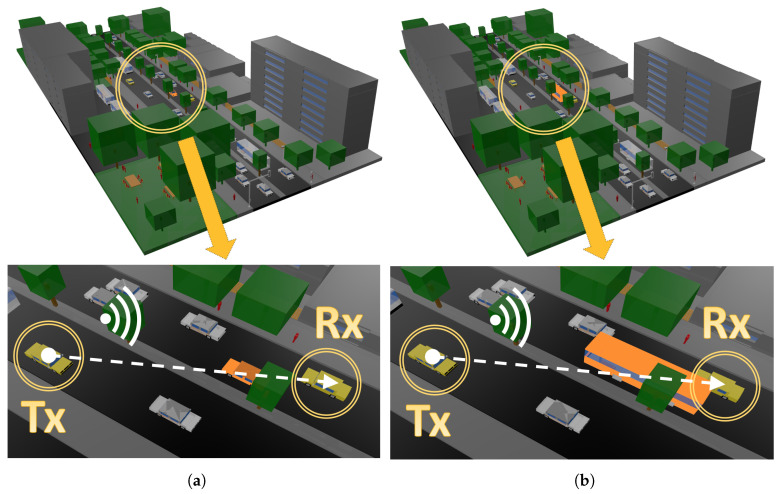
General and detailed view of the simulated 3D scenarios for both case studies: (**a**) V2V communication link with a blocking car, (**b**) V2V communication link with a bus obstruction.

This scenario has been chosen for its usefulness in terms of vehicular communication in urban environments. The presence of a median strip with abundant trees constitutes an engaging and challenging scenario for V2V communications between vehicles at different roads. In this sense, two main scenarios have been considered for simulation: first, a car obstruction in the communication link and then, a bus obstruction for the worst case study. Both modeled scenarios with their corresponding communication links analyzed cases are depicted in Figure 5.

For both scenarios, the transmitter has been placed above the left vehicle colored in yellow (see Figure 5 for reference) located in the road at 1.5 m height. Two different use case setups have been considered for simulation. On the one hand, the first configuration using an omnidirectional antenna emulating a more typical/conventional user case hardware setup with 0 dBi antenna gain and 28 GHz transmission frequency. On the other hand, an steerable antenna has been considered for the second use case setup, which can be associated with more demanding applications, such as law enforcement, ambulances, firefighters or road safety applications, where a quick response and a high demanding performance is required. The steerable antenna characteristics are the following: a beamwidth of 60 degrees, 0 dBi antenna gain and 28 GHz transmission frequency. Furthermore, the in-house 3D-RL tool allows the configuration of a Power Amplifier (PA) and a Low Noise Amplifier (LNA) in the transmitter and/or receiver link side, respectively, according to the different use case setups considered. In Table 2, the summarized simulation parameters are presented.

At the same time, for both scenarios, the receiver has been located above the right vehicle colored in yellow (see Figure 5 for reference), on the opposite road lane of the transmitter, at a distance of approximately 26 m. As has been introduced, two different scenarios have been emulated for each use case setup, considering a LOS communication obstruction by a regular car (colored in orange in Figure 5a) or a bus (colored in orange in Figure 5b). The specific characteristics of each blocking object are: the car is 4.6 m long, 1.6 m width and 1.5 m high while the bus is 11 m long, 2.7 m width and 3.45 m high. Finally, it must be remarked that all the realism of the stage has been considered for simulation, from the luminaires locations to the traffic lights, as well as the irregular people traffic, emulating real-case conditions.

### 3.3. Campaigns of Measurements

**Figure 6:**
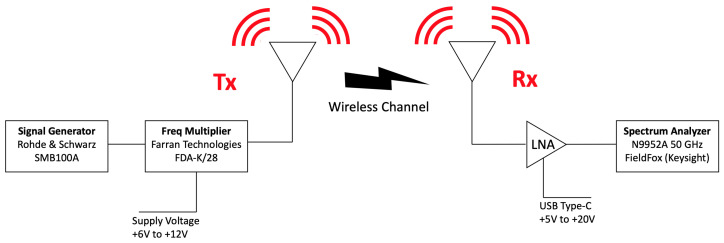
Schematic view of the deployed equipment during the campaign of measurements. Measurements were performed at 28 GHz frequency band.

**Figure 7:**
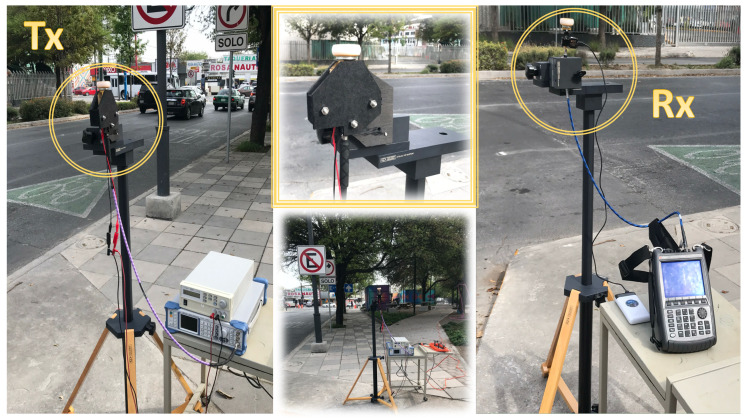
Collage of the measurement equipment used during the measurement campaign and detailed view of the transmitter system as well as the receiver composition.

An experimental campaign of measurements has been performed in the real scenario located in Monterrey, Mexico, to validate the proposed simulation algorithm. Figure 6 presents the schematic view of the deployed equipment. For the transmitting part, a signal generator SMB100A from Rohde & Schwarz up to 20 GHz has been used. A frequency multiplier FDA-K/28 from Farran Technologies has been connected to the signal generator to increase the transmitted signal up to 28 GHz. Then, a Ka-band omnidirectional antenna Model SAO-2734030345-KF-S1, from SAGE Millimeter, Inc. has been used. For the receiving part, a Ka-band omnidirectional antenna has been used, Model SAO-2734033045-KF-C1-BL from SAGE Millimeter, Inc., equipped with a low noise amplifier (LNA) of 30 dBi. Finally, the received power has been measured with a portable spectrum analyzer N9952A 50 GHZ FieldFox from Keysight Technologies. A collage with real pictures of the scenario, with the deployment of the employed equipment, as well as the surroundings of the considered scenario can be seen in Figure 7. The transmitter and receiver antennas have been placed at 1.5 m height with the aid of a tripod constructed from wood and nylon materials (AT-812 Antenna Tripod from Com-Power Corporation).

**Figure 8:**
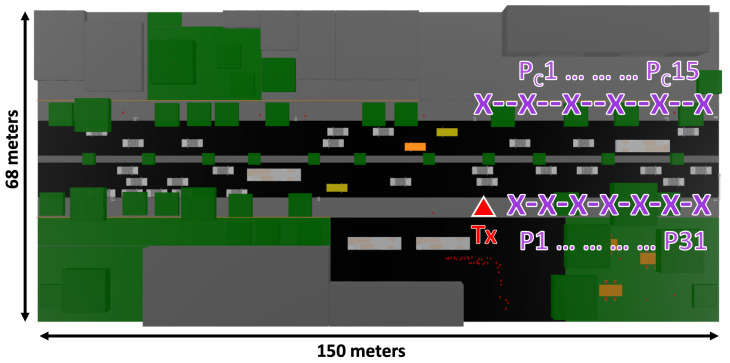
Aerial view of the measurement schematic approach followed in the measurement campaign with the transmitter location depicted, as well as the measurements points evaluated.

The campaign of measurements was performed in a common business day in the morning hours with medium vehicles’ density along the road. The transmitter antenna was placed at 1.5 m height on the sidewalk by the roadside. The specific transmitter location can be seen in Figure 8 depicted with a red triangle in the considered scenario. Measurements were performed along the same sidewalk as the transmitter, with a separation distance between them of 1.20 m (with a total of 31 points depicted as P1 to P31 in Figure 8), and across the sidewalk with a separation distance between them of 2 m (with a total of 15 points depicted as $P_{c}1$ to $P_{c}15$ in Figure 8). The spectrum analyzer was set up with a center frequency of 28 GHz and 100 MHz bandwidth, with a measurement time at each point of 60 s. Received power was measured at each point with the highest peak (Max-Hold function in the spectrum analyzer), to compare with equivalent continuous wave operation provided by the 3D-RL simulation algorithm. Please note that a streetlight was at the end of the road, so measurements were performed at the time intervals that traffic was stopped due to a red light.

## 4. Simulation Results

**Figure 9:**
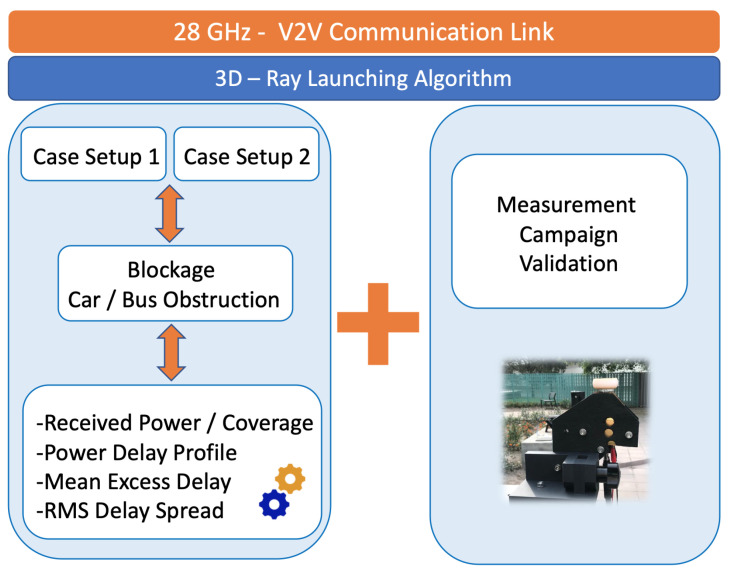
Schematic diagram of the approach presented in this work.

The following section presents the simulation results performed with the in-house 3D-RL software. As stated before, the simulated scenarios correspond to a typical urban environment and links with the obstruction by small and large vehicles (car and bus obstruction, respectively). The analysis of the received power, the Power Delay Profile (PDP), and temporal dispersion parameters such as the Mean Excess Delay (MED) and the Root-mean-squared Delay Spread (RMS DS) are shown. Figure 9 presents a schematic diagram of the 28 GHz vehicular communication link analysis presented in this work.

### 4.1. Received Power

**Figure 10:**
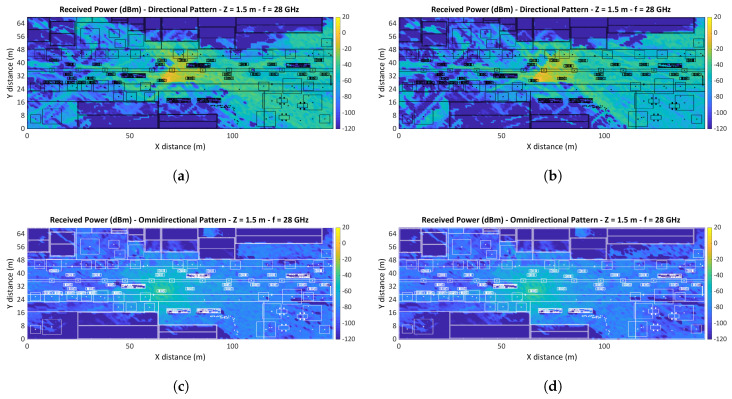
Received power (dBm) at 1.5 m height: (**a**) Directional radiation antenna pattern and bus obstruction, (**b**) Directional radiation antenna pattern and car obstruction, (**c**) Omnidirectional radiation antenna pattern and bus obstruction, (**d**) Omnidirectional radiation antenna pattern and car obstruction.

All the received power levels in dBm for the complete volume of the scenario have been collected using the in-house 3D-RL simulation tool. Figure 10 shows the horizontal planes at a height of 1.5 m for every combination (directional/omnidirectional antennas radiation pattern and car/bus link obstruction in V2V communication). Please note that the values of received power for different antennas radiation patterns planes cannot be compared to each other in numerical terms since the antenna gain differs from each other.

The coverage area associated with mmWave band communication will be reduced compared to sub 6 GHz band systems, this related to high free-space losses, poor penetration, and poor signal diffraction. This coverage area is assessed in the range of 100 to 200 m by different studies [67,68,69]. Vehicular communications are associated with low height antennas that increase blockage probabilities, and together with mmWave band communication impairments, suppose an even more aggravated effect on the coverage range. At present, there are no commercially devices dedicated to vehicular communication in the mmWave bands, thus the outage value at this point is a matter of estimation. To this end the outage value used in the simulation is −120 dB, in agreement with other studies carried out at 28 GHz for communication systems [67], considering all values lower than −120 dB as points without usable signal reception.

Figure 10c,d shows the received power in dB for the entire simulated area under an omnidirectional radiation pattern. Following the outage conditions, the signal reception throughout the avenue area in both simulation cases is approximately 98% of the total area. Taking into consideration the sidewalk as a reception area for RSU, the percentage of the area with a usable signal is reduced to 97.8%, this related to the numerous objects on the sidewalks that cause signal blocking (trees, people, lampposts). Under these conditions, the reception is achievable throughout the avenue for V2V communications, as well as on the sidewalk for V2I (RSU placed at 2–3 m height). This agrees with coverage range estimates for mmWave bands communications [67,68,69]. The areas inside buildings, as well as inside vehicles, suffer much higher losses, and in most cases, a complete signal outage due to poor signal penetration.

**Figure 11:**
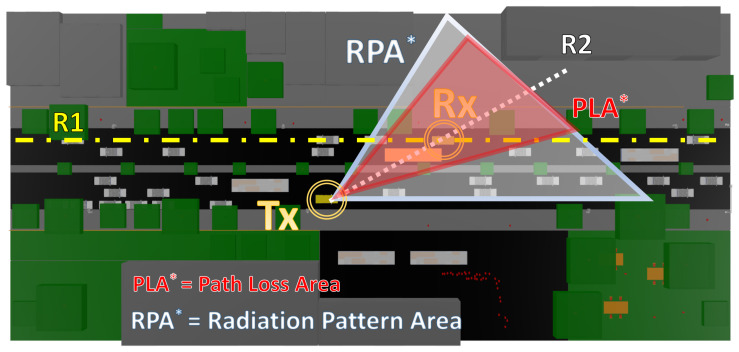
Schematic view of the simulation analysis areas with the Radiation Pattern Area, the Path Loss Area and the evaluated linear distribution radials depicted for comprehension purposes.

The use of spatial techniques such as beamforming and the application of MIMO systems promise to increase communication ranges at mmWave frequencies. Figure 10a,b shows the simulation of a V2V communication using a directional antenna. Although other areas receive useful signal power due to effects such as signal reflection and diffraction, the area of interest is limited. Connection throughout directional radiation patterns employs target tracking to establish communication. In this aspect, the main region of concern is the directional radiation pattern area (RPA) of the transmitter (see Figure 11 for reference). In this reception area, the approximate coverage, taking into account both the avenue and the sidewalk, is 99.6% in the case of the obstruction by a regular vehicle and 98% in the case of the obstruction by the bus. The reduction is linked to the shadowing by the bus, with a complete loss of the signal inside the vehicle. Thus, reinforcing the conclusion that communication in mmWave frequency bands are strongly affected by blockage.

**Figure 12:**
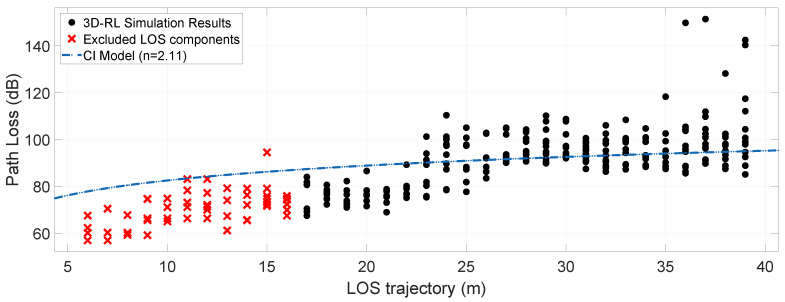
Path Loss (dB) with a car obstruction in a 17-degree area (PLA) and the mean $\bar{PLE}$ estimation for a CI free-space model.

**Figure 13:**
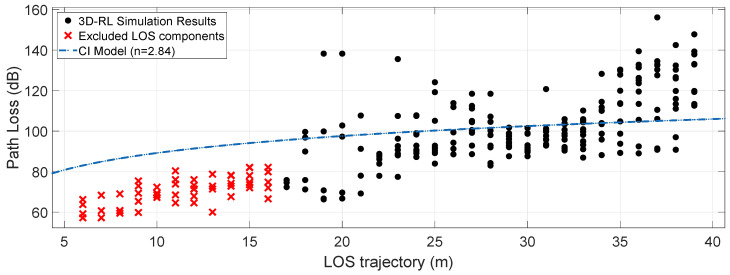
Path Loss (dB) with a bus obstruction in a 17-degree area (PLA) and the mean $\bar{PLE}$ estimation for a CI free-space model.

In outdoor wireless communications, estimating path losses is a relevant modeling parameter [70]. Signal transmission using the mmWave bands is related to substantial penetration losses by objects that usually do not cause such a sharp effect at frequencies below 6 GHz. Figure 12 and Figure 13 show the Path Loss for the simulated scenarios, considering the interposition between the transceivers of two vehicles of different sizes. Radials in the direction of the line-of-sight (LOS) corresponding to a Path Loss Area (PLA) represented in Figure 11 were used, taking into account the dimensions of the obstruction by the bus as the maximum area aperture. For this area of 17 degrees wide, one path per degree is taken, and the mean path loss exponent ($\bar{PLE}$) and mean shadow fading ($\bar{\sigma_{SF}}$) is estimated. For both objects, the distance between the transmitter and the obstruction is approximately 17 m, considering the points before this distance as LOS points.

The model that is used to adjust the path loss from the simulation results is the CI free-space model with the advantage that within its parameters, there is a frequency dependence. The equation for estimating path losses through the CI model is [69]:

(3) $$PL_{CI}\left(f,d\right)\left[dB\right]=FSPL\left(f,d\left(1m\right)\right)\left[dB\right]+10n\log\left(d\right)+\sigma_{SF}$$

where $FSPL\left(f,d\left(1m\right)\right)=20\log\frac{4\pi f}{c}$ denotes the free-space path loss in dB at a Transmitter-Receiver (TR) separation distance of 1 m at the carrier frequency *f*; *c* is the speed of light, *n* is the path loss exponent (PLE), *d* is the 3D separation distance between the transmitter and the receiver, and $\sigma_{SF}$ is the shadow fading standard deviation for large-scale signal fluctuations. The average fit of the results collected in the simulation to the CI free-space model is given in Figure 12 and Figure 13. A $\bar{PLE}$ of 2.11 and a $\bar{\sigma_{SF}}$ of 11.09 dB is obtained for the regular vehicle obstruction and a $\bar{PLE}$ of 2.84 and a $\bar{\sigma_{SF}}$ of 15.69 dB for a large dimensions vehicle obstruction (bus). Table 3 incorporates the $\bar{PLE}$ values and $\bar{\sigma_{SF}}$ of the simulated data for its fit to the CI free-space model, into a summarize simulation results table.

Obstruction by small and large vehicles have been considered in recent investigations, reporting losses between 8.2 dB and 17 dB due to the blocking by large vehicles, adding an average loss of 6 dB on top of a small blocker losses and maximum average large blocker loss of 17 dB [71]. In the simulations, received powers of −36.21 and −41.27 dBm are obtained for the scene with directional radiation patterns, in cases of small vehicle and large vehicle obstruction, respectively. These results represent an addition in the path losses of a long vehicle over a small one of approximately 5 dB. In the case of the omnidirectional pattern, the power received at the target is −72 dBm and −85.04 dBm for the small vehicle and the bus respectively, corresponding to a higher 13 dB loss in the case of the bus obstruction. As a result, according to the simulation results, the difference in path loss that a large obstacle overlaps over a small one ranges between 5 and 13 dB, which have a good agreement with observations made in the literature [71].

### 4.2. Power Delay Profile (PDP)

**Figure 14:**
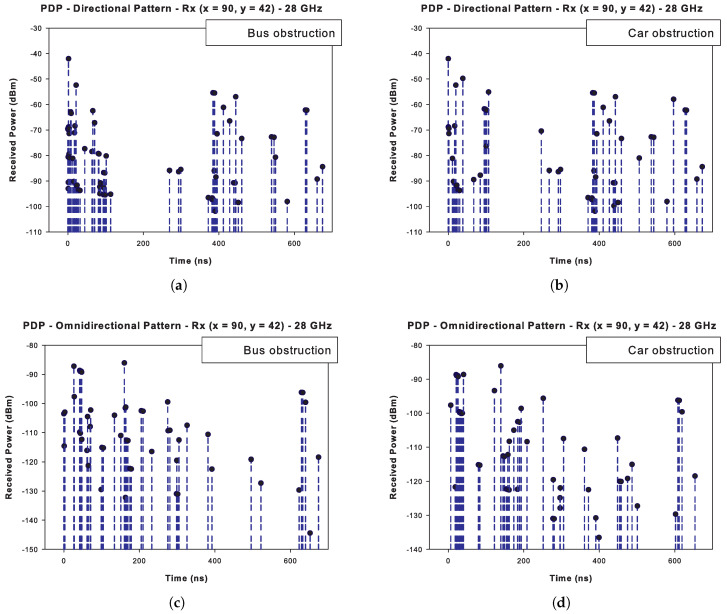
Power Delay Profile (dBm) at 1.5 m height: (**a**) Directional radiation antenna pattern and bus obstruction, (**b**) Directional radiation antenna pattern and car obstruction, (**c**) Omnidirectional radiation antenna pattern and bus obstruction, (**d**) Omnidirectional radiation antenna pattern and car obstruction.

The PDP is one of the most important parameters to characterize multipath propagation in complex environments. From its information, relevant parameters are derived to describe the frequency selectivity of the channel such as the RMS DS or the MED. Figure 14 shows the PDP for the selected frequency (28 GHz); the values are obtained for the receiver yellow car located on the opposite road lane of the transmitter, at a distance of approximately 26 m (see Figure 5 for reference). Figure 14a,b correspond to the directional antenna radiation pattern, and Figure 14c,d correspond to the omnidirectional antenna radiation pattern under the two scenarios conditions.

Although the 3D-RL simulations does not theoretically impose a limit in the dynamic range (DR), in this paper a 60 dB DR is used, an in between value within some DR of 240 dB [72], 178 dB [69], and 30 dB [71] found in the literature from experimental studies. This limitation in the DR value also provides a realistic approach, so achieving results more consistent with the restrictions and performance limits of hardware elements, as demonstrated in [65]. It is worth noting from the figure, the marked disappearance of cluster paths in the directional pattern cases compared to those in the omnidirectional pattern cases. This is caused because the directional beam only uses a spatial sector for transmission, and as a result, fewer reflected/diffracted paths find their way to the receiver.

As stated, the omnidirectional antenna transmission is simulated with lower gain power than the directional pattern. Figure 14c shows the PDP when a large vehicle (bus) is found in the LOS path between the transceivers. It is noteworthy that some of the first resolvable paths are heavily affected compared to their counterpart (Figure 14d), a small vehicle (car). This is closely related to the poor penetration of signals at mmWave frequencies bands. The decrease in received power of some of the first-order resolvable paths is especially notable. This corresponds to rays of shorter paths travel distances, consequently strongly affected by the obstruction. The rest of the multipath components are less affected as they are related to high delays and, consequently, greater distances traveled, thus possibly being related to paths away from the obstruction.

For the directional pattern, Figure 14a,b depict the PDPs of the bus obstruction and a car obstruction, respectively. Similarly, there is a strong influence of the large obstacle over the first-order paths. Other feature defined in the directional pattern PDP in both situations is the grouping of the different multipath components into regional clusters, in accordance with the spatial consistency of deterministic approaches.

An important multipath parameter that can be extracted from the PDP is the MED, this parameter is determined by Equation (4) and is defined as the first moment of the PDP [73].

(4) $$MED=\frac{\sum_{k}P\left(\tau_{k}\right)\tau_{k}}{\sum_{k}P\left(\tau_{k}\right)}$$

where $P(\tau_{k})$ is the power (dB) of the *k*th path, and $\tau_{k}$ is the delay of the *k*th path. Table 3 shows the mean excess delay values at the receiver point for both radiation patterns and both obstructed simulations. In the simulation with an omnidirectional pattern, the MED registers values of 245.7 ns for the scenario with a regular car and 227.8 ns for the bus obstruction. Similarly, in the scenarios with a directional pattern, values of 294.6 ns and 221.5 ns are found for the obstruction by small and large vehicles, respectively. Although the values cannot be compared between radiation patterns (due to the difference in transmission power gain), the increase in MED in the cases of obstruction by a small vehicle to the bus simulations are noticeable. This is related to the substantial attenuation received by first-order paths when impacting a large object.

### 4.3. RMS Delay Spread (RMS DS)

One of the most relevant parameters that describe the temporal dispersion of the multipath channel is the RMS DS, closely related to other important parameters such as the coherence bandwidth. In some studies carried out in vehicular communication for sub 6 GHz frequency bands, it is concluded that when an obstruction occurs, the RMS DS can suffer an increase in values up to 400 ns [74,75], recently this conclusion has been supported by studies carried out in the mmWave frequencies, concluding that when there is a block in the LOS, an increase in the RMS DS is observed [71].

**Figure 15:**
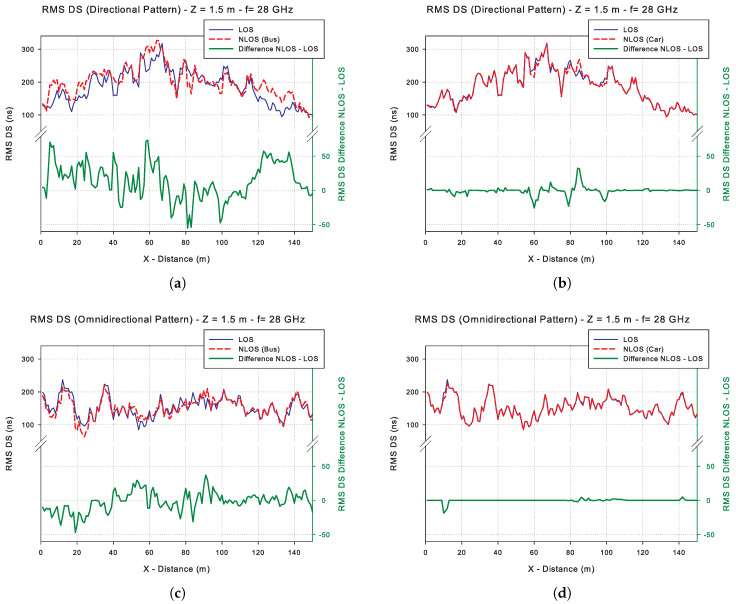
RMS DS simulation results for a radial R2 in the receiver trajectory: (**a**) Directional radiation antenna pattern and bus obstruction, (**b**) Directional radiation antenna pattern and car obstruction, (**c**) Omnidirectional radiation antenna pattern and bus obstruction, (**d**) Omnidirectional radiation antenna pattern and car obstruction.

Figure 15 shows a radial (trajectory R2 represented in Figure 11) in the path of the receiving vehicle along the avenue, where the RMS DS values are plotted at each discrete point of the simulation. The X-axis corresponds to the distance in meters from the radial on the avenue. The RMS DS values for these points are plotted in two instances: the upper part corresponds to the values of two simulations, one with obstruction (NLOS) and one without obstruction (LOS). The bottom of each graph corresponds to the calculated difference between the RMS DS values when an obstruction exists and the LOS values. In all the simulated cases, the RMS DS values range from approximately 100 ns to 300 ns with local minima in some cases of 63 ns and maxima of 326 ns.

Table 3 shows a summary of the RMS DS values in the simulated scenarios, where it can be seen that at the receiver area there is an increase in RMS DS with respect to the simulations without obstructions. In the case of the omnidirectional radiation pattern, there is an increase of 3.24 ns and 21.14 ns for the cases of obstruction by small vehicles and large vehicles, respectively. In the case of communication with the directional antenna, there are increases of 2.16 ns and 3.74 ns respectively, a decrease in the temporal dispersion to its omnidirectional counterpart, especially in the case of obstruction by the bus. It has been reported that the RMS DS decreases with the use of narrow beams, but more measurements in this regard should be considered [71]. Outside the receiver’s area, the RMS DS values in some cases exceed 50 ns over the LOS simulation, characterizing the RMS DS variable with a high dispersion, linked to the type of obstruction that is used. Table 3 shows the variance ($\sigma_{R1}^{2}$ (RMS DS)) of the difference between the simulations (NLOS-LOS) along R1 trajectory (see Figure 11 for reference). It is worth noting the increase of $\sigma_{R1}^{2}$ for the cases of obstructions by a large vehicle, closely related to the increase in the number of reflected paths on the bus surface.

## 5. Measurements Results

**Figure 16:**
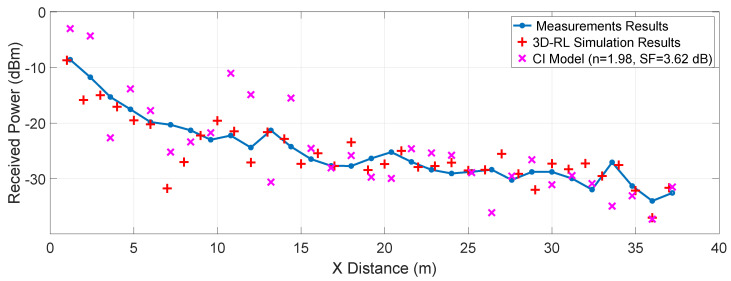
Measurement results for $P_{1}$ to $P_{31}$ locations (see Figure 8 for reference), 3D-RL simulation results for these spatial positions, and a CI free-space model approximation (n=1.98 $\sigma_{SF}=3.62$).

To validate previous simulation results, several measurement points were collected in the real scenario presented in the Section 3.3, with the previous explained deployed equipment. Figure 16 exhibits the results gathered throughout the measurements campaign, in markers from $P_{1}$ to $P_{31}$ as represented by the layout in Figure 8. The empirical data is represented in circles joined by linear interpolation, where every point is spaced by 1.2 meters, starting from 1.2 to 37.2 m in the x-axis. The 3D-RL results of the simulation carried out on the virtual stage, under the conditions of the measurement campaign, are illustrated in the same figure by red “+” symbols. The root-mean-square error (RMSE) between measurements and simulation is 2.91 dB, showing good agreement between simulations and the empirical data.

To compare with related work in the literature, a study carried out by Longhe Wang et al. [44] based on the characterization of the wireless channel in a V2I scenario, has been considered. The work analyzes different scenarios, where the fitted values for a CI free-space model are obtained in terms of its PLE and shadow fading ($\sigma_{SF}$). Using the PLE *n* = 1.98 and a $\sigma_{SF}=3.62$ dB, which is the best fit determined in their investigation for the LOS cases, the received signal power estimation is plotted in Figure 16 following the CI free-space model. The RMSE of this estimate is 5.02 dB, an error greater than that of the 3D-RL simulation presented in this work. This shows the close relationship of the PLE and the $\sigma_{SF}$ with the specific environment that it intends to estimate. The scenario in which the measurements and the 3D-RL simulation occur presents a favorable environment to the wave propagation, with abundant scatterers on both sides of the LOS path. Large metal elements are also on the sides, influencing better signal reception throughout the path. As a result, the use of PLE based on generic scenarios can lead to estimation errors, while deterministic simulations such as RL can better demonstrate the phenomena that characterize a given scenario.

**Figure 17:**
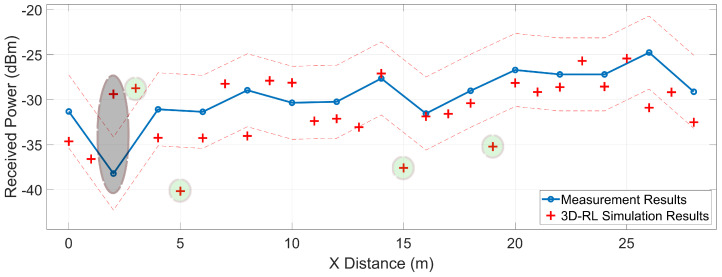
Measurement results for the opposite sidewalk ($Pc_{1}$ to $Pc_{15}$) and 3D-RL simulation results for these spatial locations.

The second set of measurements were taken at the opposite sidewalk, marks from $Pc_{1}$ to $Pc_{15}$ measurements points in Figure 8. Figure 17 shows the collected data, where each point is spaced by 2 m, starting from x=0 ($Pc_{1}$) to x=28 ($Pc_{15}$). Please note that Figure 8 is for representation and it is not render at the exact scale. These measurements emulate a V2I (RSU) communication when the latter is on the other side of the avenue.

The corresponding 3D-RL software simulation results are plotted together with the empirical values, where the 3D-RL data points are denoted by red “+” symbols. The RMSE between simulation and the experimental measurements is 4.06 dB, being higher in this case because the measurements were made with different vehicle distributions at every point ($Pc_{1}$ to $Pc_{15}$). Although many of the objects found during the measurement campaign are carefully represented in the simulation, differences between the distribution of the vehicles increase the error margin.

Some of the points with a greater margin of error are highlighted by gray and light green shapes. The gray ellipse corresponds to the measurement made at $Pc_{2}$ and its representation in the simulation environment. At this spatial point a value of −38 dB is registered in the empirical measurement and −29 dB in the simulation. The point $Pc_{2}$ corresponds to a measurement made under NLOS conditions due to obstruction by a bus. The difference between the simulation and the empirical value is 9 dB, agreeing with the estimations made by the obstruction of a large vehicle in the simulation section. The data points signaled by light green circles correspond to simulations results that constitute some of the greater sources of errors. These points do not correspond to any actually measured location, therefore the error is an estimation over the lineal interpolation of the measurements. These errors are also related to the differences in vehicle distributions between the simulated scenario and the real one. The dashed red lines in Figure 17 presents the mean shadow fading area caused by the different vehicle distributions along the road.

## 6. Conclusions

In this paper, a wireless communications channel in the mmWave frequency band (28 GHz) for vehicular environments is characterized based on the assistance of a 3D-RL software and empirical data. The proposed simulation methodology enables the consideration of full volumetric scenario distribution and the consideration of detailed scatterer distributions, enabling the analysis of scenario as well as traffic conditions. Two different use cases have been considered: the first one with an omnidirectional antenna for a more typical/conventional user hardware configuration, and the second one using a steerable antenna, which can be associated with high demanding performance applications. For both cases, an V2V link obstruction with vehicles of different sizes (car vs bus) has been analyzed. Results from simulation and measurements have a good match and agree with the statement that vehicular communication in the mmWave frequency band is plausible in ranges from 100 to 200 m.

It is observed that the inclusion of vehicles of distinctive sizes causes different effects on the channel, showing that the received power at the target is affected approximately between 5 to 13 dB when a large dimension vehicle is between the LOS. In our simulations, a data fit for the CI free-space model exhibits a $\bar{PLE}$ of 2.11 and a $\bar{\sigma_{SF}}$ of 11.09 dB for a small dimension obstacle and a $\bar{PLE}$ of 2.84 and a $\bar{\sigma_{SF}}$ of 15.69 dB for an obstruction due to a large dimensions vehicle (bus). Other parameters that are affected by the size of the obstruction are the MED and the RMS DS. In the case of the MED, an increase in its nominal value is observed in the simulations with a small obstruction (regular car) with respect to the bus scenarios, this related to the high attenuation that first-order rays receive by the bus obstruction. In the case of RMS DS, there is a decrease in the directional pattern scenarios, an estimation also pointed out in other studies.

As a complement to the simulations, a measurement campaign was carried out in the real scenario, where the results of these measurements show an agreement with the 3D-RL simulations. The RMSE of the simulations with respect to the measurement campaign is 2.94 dB, a better fit than the use of a CI model presented in the literature for similar scenarios with an estimated PLE of n=1.98 and a shadow fading of $\sigma_{SF}=3.62$ dB [44]. Thus, revealing the close relationship of these parameters with specific scenarios, features that are taken into consideration in deterministic approaches. The RMSE for the measurements performed on the other side of the avenue is 4.05 dB, where the increase in error is related to the different vehicle distributions at every point of the measurements. Future work will be directed to the analysis of this margin for dynamic traffic flow. The proposed methodology can be adapted to consider any variation in terms of network topology, location and configuration of the employed transceivers, characteristics of the surrounding environment or variable node density.

## Figures and Tables

**Table 1:** RT/RL approaches for mmWave frequency bands.

REF	Type	Environment	Channel Metrics	Frequency	Year
[36]	-	Indoor	PDP, PAS	60 GHz	2017
[37]	Cellular	Urban Microcell	PL exponent	28 GHz	2018
[38]	-	Inside a Bus	RMS DS, PL	60 GHz	2018
[39]	Cellular	Urban	PL exponent, RMS DS	72 GHz	2013
[40]	V2I	Urban	PL, RMS DS	28 GHz	2018
[41]	HST	Several	PL, RMS DS, PDP, CT	25.25 GHz	2018
[42]	HST	Rural railway	PL, RMS DS	28 GHz	2019
[43]	V2I	Urban/Highway	KF, RMS DS, AS	60 and 21.6 GHz	2020
[44]	V2I	Urban	PDP, KF, RMS DS, AS	28 GHz	2019

Power Delay Profile (PDP), Power angular spectrum (PAS), root-mean-square Delay Spread (RMS DS), Path Loss (PL), Rician K-factor (KF), Angular Spread (AS), Coherence Time (CT), High-Speed Trains (HST).

**Table 2:** RL algorithm simulation parameters.

	Setup Case 1	Setup Case 2
Frequency	28 GHz	
TX Power	10 dBm	
TX Antenna type/Power Amplifier/Gain	Monopole/0 dBi/0 dBi	Steerable 60°/20 dBi/0 dBi
RX Antenna type/Low Noise Amplifier/Gain	Monopole/20 dBi/0 dBi	Steerable 60°/20 dBi/0 dBi
3D-RL Angle Resolution (Δϕ/Δθ)	1°	0.25°
Number of reflections	6	
Scenario size/Unitary volume analysis	150 m × 68 m × 22 m/1 m × 1 m × 1 m	

**Table 3:** Simulation Results.

	Omnidirectional Pattern		Directional Pattern	
	Car Obstruction	Bus Obstruction	Car Obstruction	Bus Obstruction
$\bar{PLE}$	-	-	2.11	2.84
$\bar{\sigma_{SF}}$ (Path Loss)	-	-	11.09 dB	15.69 dB
Mean Excess Delay	245.7 ns	227.8 ns	294.6 ns	221.5 ns
RMS DS difference (NLOS-LOS )	3.24 ns	21.14 ns	2.16 ns	3.74 ns
$\sigma_{R1}^{2}$ (RMS DS)	2.35 ns	14.25 ns	6.23 ns	26.44 ns
